# State of open science in cancer research

**DOI:** 10.1007/s12094-024-03468-7

**Published:** 2024-04-18

**Authors:** Cristina Rius, Yiming Liu, Andrea Sixto-Costoya, Juan Carlos Valderrama-Zurián, Rut Lucas-Dominguez

**Affiliations:** 1 UISYS Group, Department of History of Science and Information Science, Faculty of Medicine and Dentistry, University of Valencia, Valencia, Spain; 2 Unit associated with the Interuniversity Institute for Advanced Research on the Evaluation of Science and the University (INAECU) UC3M-UAM, Madrid, Spain; 3 Spanish National Center for Cardiovascular Research (CNIC), Madrid, Spain; 4 CIBERCV, Madrid, Spain; 5 Department of Social Work and Social Services, Faculty of Social Sciences, Universitat de València, Valencia, Spain; 6 CIBERONC, Valencia, Spain

**Keywords:** Cancer research, Open science, Open access, Data sharing, Scientific impact

## Abstract

**Purpose:**

This study has been focused on assessing the Open Science scenario of cancer research during the period 2011–2021, in terms of the derived scientific publications and raw data dissemination.

**Methods:**

A cancer search equation was executed in the Science Citation Index-Expanded, collecting the papers signed by at least one Spanish institution. The same search strategy was performed in the Data Citation Index to describe dataset diffusion.

**Results:**

50,822 papers were recovered, 71% of which belong to first and second quartile journals. 59% of the articles were published in Open Access (OA) journals. The Open Access model and international collaboration positively conditioned the number of citations received. Among the most productive journals stood out *Plos One*, *Cancers*, and *Clinical and Translational Oncology*. 2693 genomics, proteomics and metabolomics datasets were retrieved, being Gene Expression Omnibus the favoured repository.

**Conclusions:**

There has been an increase in oncology publications in Open Access. Most were published in first quartile journals and received higher citations than non-Open Access articles, as well as when oncological investigation was performed between international research teams, being relevant in the context of Open Science. Genetic repositories have been the preferred for sharing oncology datasets. Further investigation of research and data sharing in oncology is needed, supported by stronger Open Science policies, to achieve better data sharing practices among three scientific main pillars: researchers, publishers, and scientific organizations.

**Supplementary Information:**

The online version contains supplementary material available at 10.1007/s12094-024-03468-7.

## Introduction

The impact of cancer on individuals, society, and the economy is significant. By 2040, the worldwide burden of cancer will rise to 30 million cases, with the largest increases in low- and middle-income countries [1]. Globally, it is estimated that there is a prevalence of cancer at 5 years’ post-diagnosis of more than 44 million. In 2020, prostate and breast cancer ranked as the leading diagnoses in men and women, respectively, while lung cancer followed with 2.2 million cases [2]. The European Cancer Information System (ECIS) has reported a total of 2.74 million cancer patients in 2022, representing a 2.3% increase in new cancer cases compared to 2020 [3]. In Spain, over 270,000 people are diagnosed each year [4], concretely in 2023, an estimated 158,544 cases of cancer are expected to occur in men and 12,715 in women [5].

To address this situation, advances in oncology are ushering in a new era of personalized and precise medicine, transforming everyday cancer care. However, unleashing the full potential of these approaches requires sound policies to ensure their regular application in patient care, including changes and new practices in science researching directives. To contribute, the Open Science (OS) movement, whose impact on science has been remarkable in the last two decades, offers a series of useful and necessary practices to accelerate the publication and dissemination of scientific results. Starting with the Berlin Declaration on Open Access (OA) [6], today is commonly accepted by institutions, funders, and publishers that the open access contributions must necessarily include, along with the article published in OA, also the primary data and their metadata [7, 8]. Thus, after an initial phase in which the demand for publicly funded research focused on the OA publication of scientific papers, the practice of sharing the raw data is now widely recognized as a means of ensuring honesty and robustness through its role in accountability and its ability to replicate experiments, as well as being cost-effective through the re-use and improvement of existing data [9]. Particularly in cancer research, institutions such as the National Cancer Institute [10] explicitly state that “improved treatment options for cancer patients will result when researchers share their data widely with investigators in the research community”, along with Cancer Research UK [11], which directly advocates a culture of sharing research data for re-use across sectors. Similarly, the Lancet Oncology Commission “European Groundshot-addressing Europe’s cancer research challenges”, highlights the importance of “collaborative research through data sharing is essential to ensure rapid improvement in cancer care, from diagnosis to therapeutic application” [12].

Considering the significance and promptness of effective cancer research dissemination, as well as the value provided by Open Science resources such as data sharing and open access papers, this study aims to accomplish the following objectives: i) to analyse the evolution of oncology research with Spanish participation in terms of articles and research data production during the decade of 2011–2021 and ii) to assess the impact on the scientific literature of open published articles in terms of citations, journal impact factor and scientific collaborations; and the characteristics of the deposited research data through their repositories and thematic categories.

## Methods

We collected scientific publications in the field of cancer from the Science Citation Index-Expanded (SCIE) database of the Web of Science (WoS) for the period 2011–2021, signed by at least one Spanish institution. Documents were retrieved using a search equation conformed by search terms representative of cancer along with the different typologies described by the NCI cancer types (for example sarcoma, glioblastoma or leukaemia) combined with those papers published by journals belonging to the “Oncology” category of the SCIE (see Supplementary file 1). Finally, a total of 50,822 scientific documents (42,326 articles and 8496 reviews) were selected. The following variables were extracted from each document in an Access database: title, journal, publisher, year, authors, international collaboration, number of citations and open access publication route, following the classified model from WoS description: Green Accepted, Green published, Free to read (Bronze), Gold hybrid, and Gold [13]. Green submitted filter was excluded since it refers to documents that have not undergone peer review.

To study the impact of the journals, the quartiles (Q) extracted based on the JIF of the WoS categories from Journal Citation Reports database (JCR) in the respective year of publication were used. To assess the impact of the papers in the scientific literature, the total number of citations received for the articles was calculated referring to the weighted citations: number of citations received/years since publication.

To describe the dissemination of datasets derived from the oncology studies developed by at least one Spanish institution, between 2011–2021, a bibliographic search was performed in the Data Citation Index (DCI) to obtain an overview in terms of thematic categories, cites and repositories used.

A descriptive analysis of the variables was performed to obtain the frequencies and percentages. All charts, tables and figures were created using Microsoft Excel. VOSviewer software was used to represent the authorship networks related to OA publications and datasets, after normalizing the signatures and including those authors who had at least 5 documents retrieved for the period 2011–2021 and were signed by a maximum of 1000 authors per article.

## Results

### Evolution of cancer publications and their different access routes

**Fig. 1:**
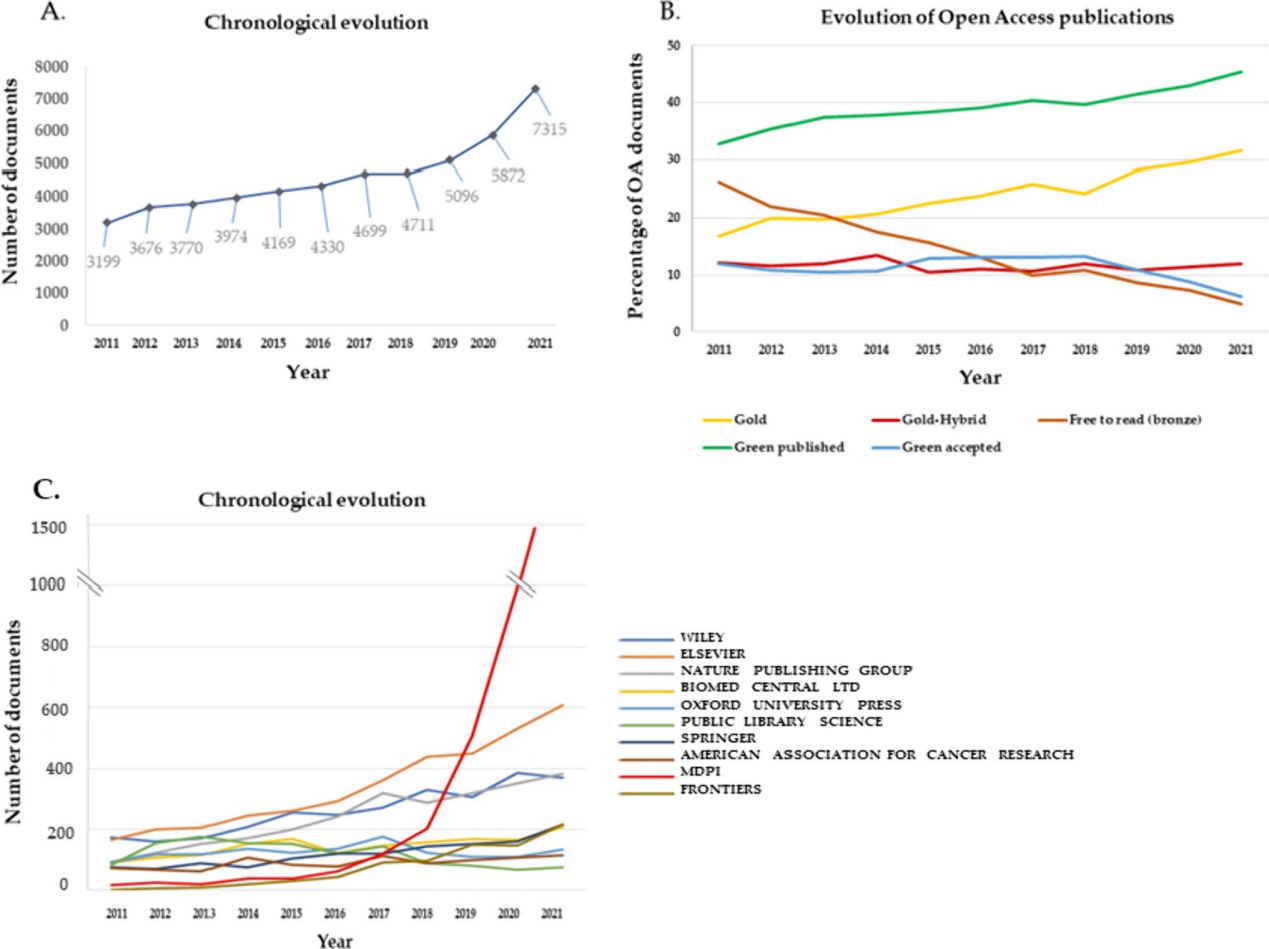
Chronological evolution of **a** scientific publications; **b** Open Access model of cancer publications developed by at least one Spanish institution during the period 2011–2021; and **c** trends of the top ten publishers related to the Open Access articles in cancer

The analysis of the 50,822 cancer publications from 2011 to 2021 produced by at least one Spanish institution showed an increasing trend over the period studied, with more than double the number of documents in 2021 (7315) compared to 2011 (3199). Specifically, half of all documents published during the period 2011–2021 were produced in the last 5 years (2017–2021) (Fig. 1a). Regarding the publication model for research communication, 41% were non-OA and 59% were OA papers. The detailed evaluation of total Open Access publications which ascended to 75% of articles in 2021 highlighted Green (45.3%) and Gold (31.7%) publishing as OA dominant routes. In 2011, only 42.7% of publications adhered to the OA model, mostly opting for the Green published (32.8%) and Free to read (26.3%) alternatives (Fig. 1b). A more in-depth analysis was carried out to find out the trends of the 10 top publishers in terms of open access articles in cancer in the studied period (2011–2021) (Fig. 1c). Since 2017 MDPI has developed an exponential growth, publishing in 2021 more than twice than Elsevier (second position), followed far behind by the other eight top publishers.

### Analysis of the impact of cancer publications in Open Access

**Fig. 2:**
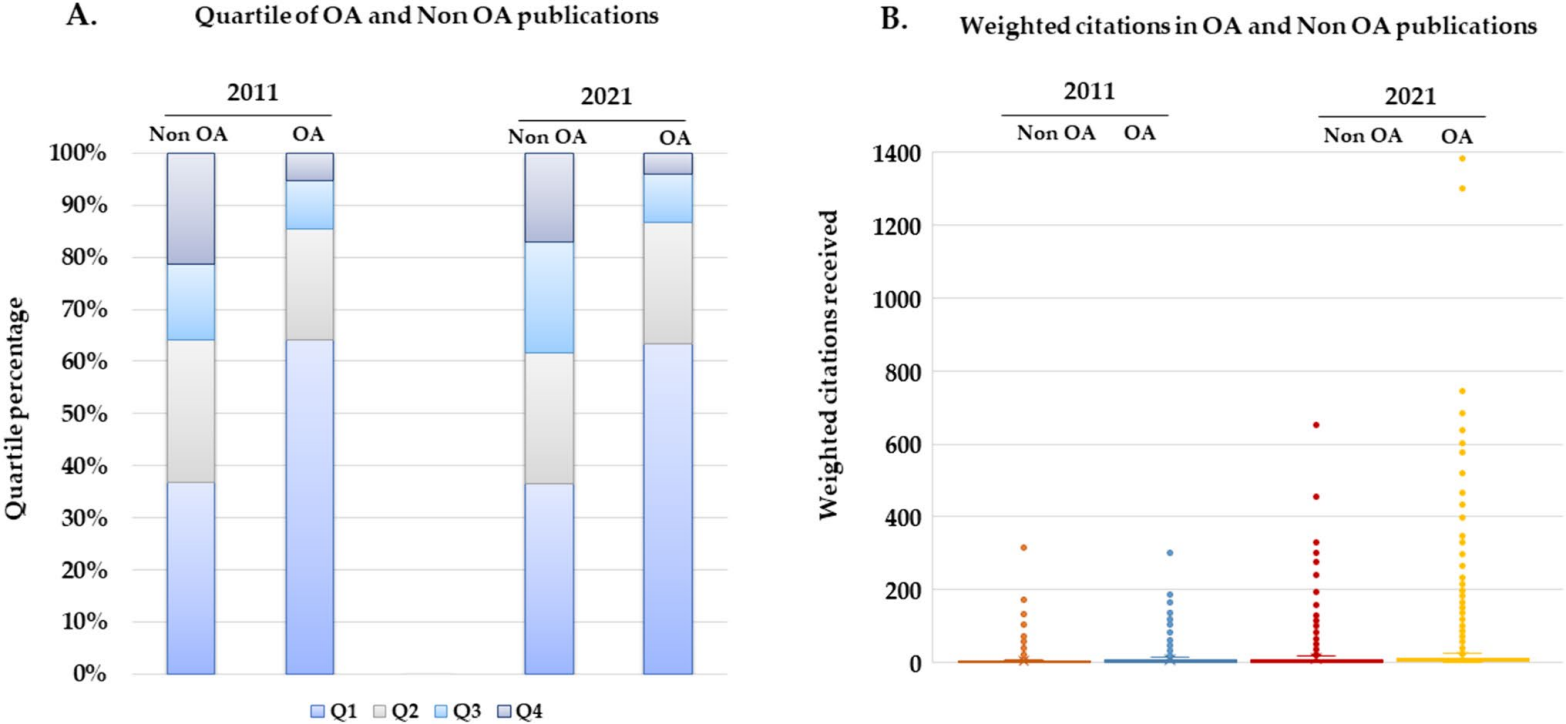
Chronological evolution of the impact of cancer publications in OA and non-OA journals measured **a** through the analysis of JIF quartiles of journals and **b** weighted citations received by the papers

Of the 50,822 articles, 49% were published in journals belonging to the quartile 1 (Q1), 22% in Q2, 13% in Q3 and 9% in Q4, while 7% were not placed in any quartile because they did not have JIF in the year of publication. Figure 2 describes the evolution of the publications in the period studied and their distribution by quartiles based on the JIF category ranking. The greatest percentage increase is recorded between 2011 and 2021 in publications in Q1 (132.7%) and Q2 (143.5%) (Supplementary Fig. 1a). The analysis of the papers published in OA (29,961) and their distribution by JIF quartiles shows that the majority are in Q1 journals (63% in 2011 and 64% in 2021) while the papers published in non-OA journals that are positioned in Q1 are 36% both in 2011 and 2021 (Fig. 2a).

Concerning the analysis of citations of cancer papers, the average weighted citations received showed a rising trend over the evaluated years (Supplementary Fig. 1b). When comparing the weighted citations received according to the OA way, it was observed that articles published in OA in both 2021 and 2011 received a greater number of citations than those published in the non-OA modality (Fig. 2b).

With regard to the analysis of the top-producing scientific journals, it is worth noting that 18.4% of all retrieved papers were published in the 20 most productive journals, and 13 of the 20 journals are indexed in Q1 of the JIF category of the JCR. This ranking is headed by the journals *PLoS One, Cancers* and *Clinical and Translational Oncology*, with Oncology standing out as the predominant WoS category (Table 1).

**Table 1:** Top 20 most productive journals on cancer research conducted by at least one Spanish institution between 2011 and 2021

Journal	Number of documents	Web of Science categories	JIF^a^
PLOS ONE	1066	Multidisciplinary Sciences	Q2
CANCERS	908	Oncology	Q2
CLINICAL & TRANSLATIONAL ONCOLOGY	886	Oncology	Q3
SCIENTIFIC REPORTS	643	Multidisciplinary Sciences	Q2
INTERNATIONAL JOURNAL OF MOLECULAR SCIENCES	550	Biochemistry & Molecular Biology; Chemistry, Multidisciplinary	Q1; Q2
ONCOTARGET	543	Oncology	Q1
ANNALS OF ONCOLOGY	527	Oncology	Q1
CLINICAL CANCER RESEARCH	445	Oncology	Q1
JOURNAL OF CLINICAL ONCOLOGY	418	Oncology	Q1
INTERNATIONAL JOURNAL OF CANCER	410	Oncology	Q1
ACTAS UROLOGICAS ESPANOLAS	376	Urology & Nephrology	Q4
EUROPEAN JOURNAL OF CANCER	364	Oncology	Q1
BLOOD	330	Hematology	Q1
LANCET ONCOLOGY	301	Oncology	Q1
CIRUGIA ESPANOLA	297	Surgery	Q3
NATURE COMMUNICATIONS	294	Multidisciplinary Sciences	Q1
LEUKEMIA	268	Oncology; Hematology	Q1; Q1
BMC CANCER	266	Oncology	Q2
BRITISH JOURNAL OF CANCER	261	Oncology	Q1
CANCER RESEARCH	221	Oncology	Q1

^a^JIF: Journal Impact Factor category ranking related to JCR edition 2022

**Fig. 3:**
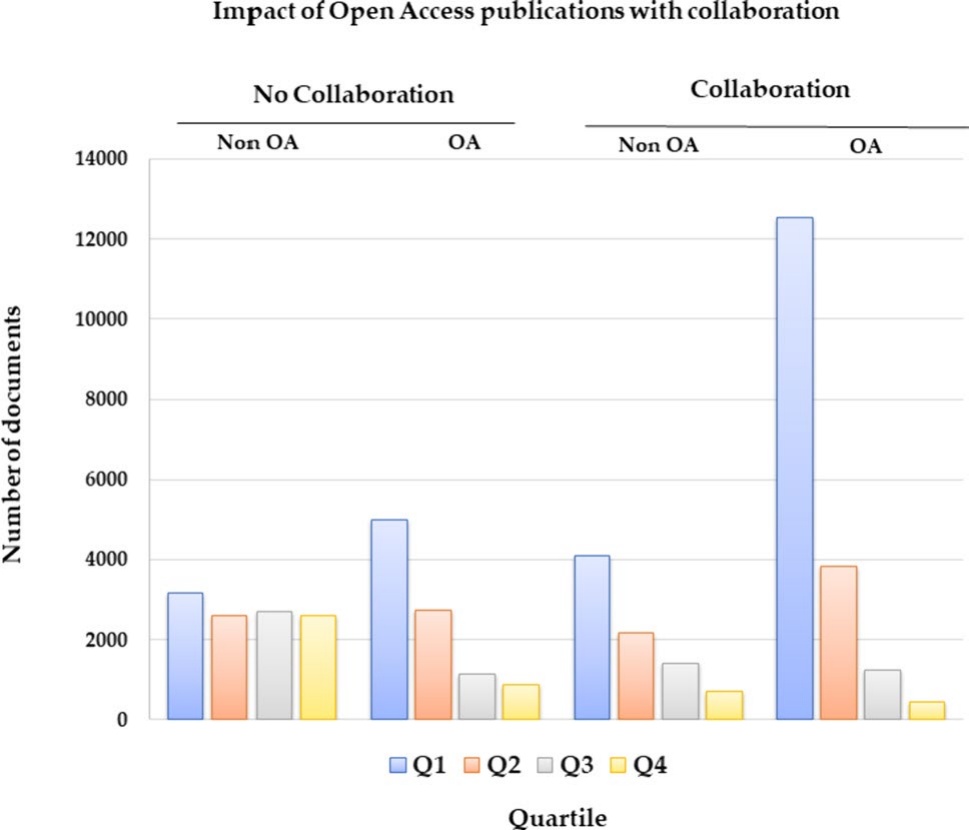
Analysis of the relationship between quartiles (Q) of journals in which OA papers have been published and international collaboration

To complete the overview of the situation of oncology research, we examined international collaboration during the period 2011–2021 in relation to the distribution of JIF quartiles of journals. Figure 3 illustrates a favourable correlation among international collaboration, Open Access publishing, and publishing in the top quartile journals.

**Fig. 4:**
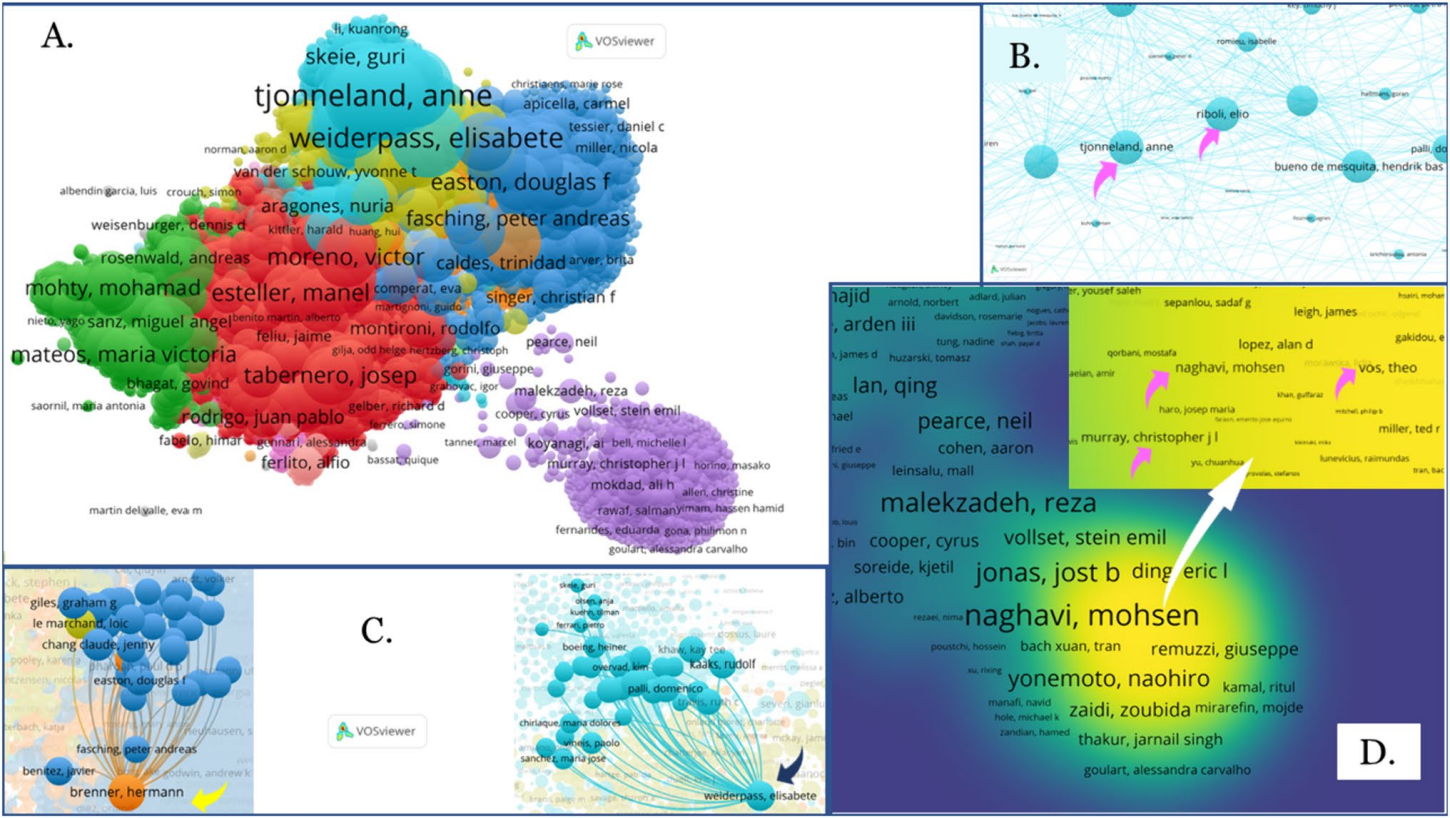
Collaborative network of authors on oncology publications (2011–2021) in the OA route. **a** General visualization. **b** Representation of the authors with the highest number of publications. **c** Distribution of the authors with the highest number of collaborations. **d** Visualization of density according to the number of citations of the authors

Further analysis identified the authors of the 29,961 papers published in OA. Figure 4a shows the 176,101 authors with at least five published papers (2011–2021) and a maximum of 1000 authors per publication, with Tjonneland, Anne standing out with 456 papers in which she collaborated with 1.489 authors (Fig. 4b). Riboli, Elio and Tumino, Rosario published 450 and 444 papers, respectively; and were linked to 1908 and 1617 authors each other (Fig. 4b). Despite not having the highest number of publications, Brenner, Hermann (n_DocBrenner_ = 189) and Weiderpass, Elisabete (n_DocWeiderpass_ = 348) were associated with more authors (n_LinkBrenner_ = 2962, n_LinkWeiderpass_ = 2345) (Fig. 4c). In relation to the researchers with the greatest scientific impact, evaluated according to the citations received, Fig. 4d shows the number of citations from the density, highlighting the authors Naghavi, Mohsen (48,518 citations), Vos, Theo (47,916 citations) and Murray, Christopher (45,988 citations).

### Analysis of the availability of raw data in cancer research

**Fig. 5:**
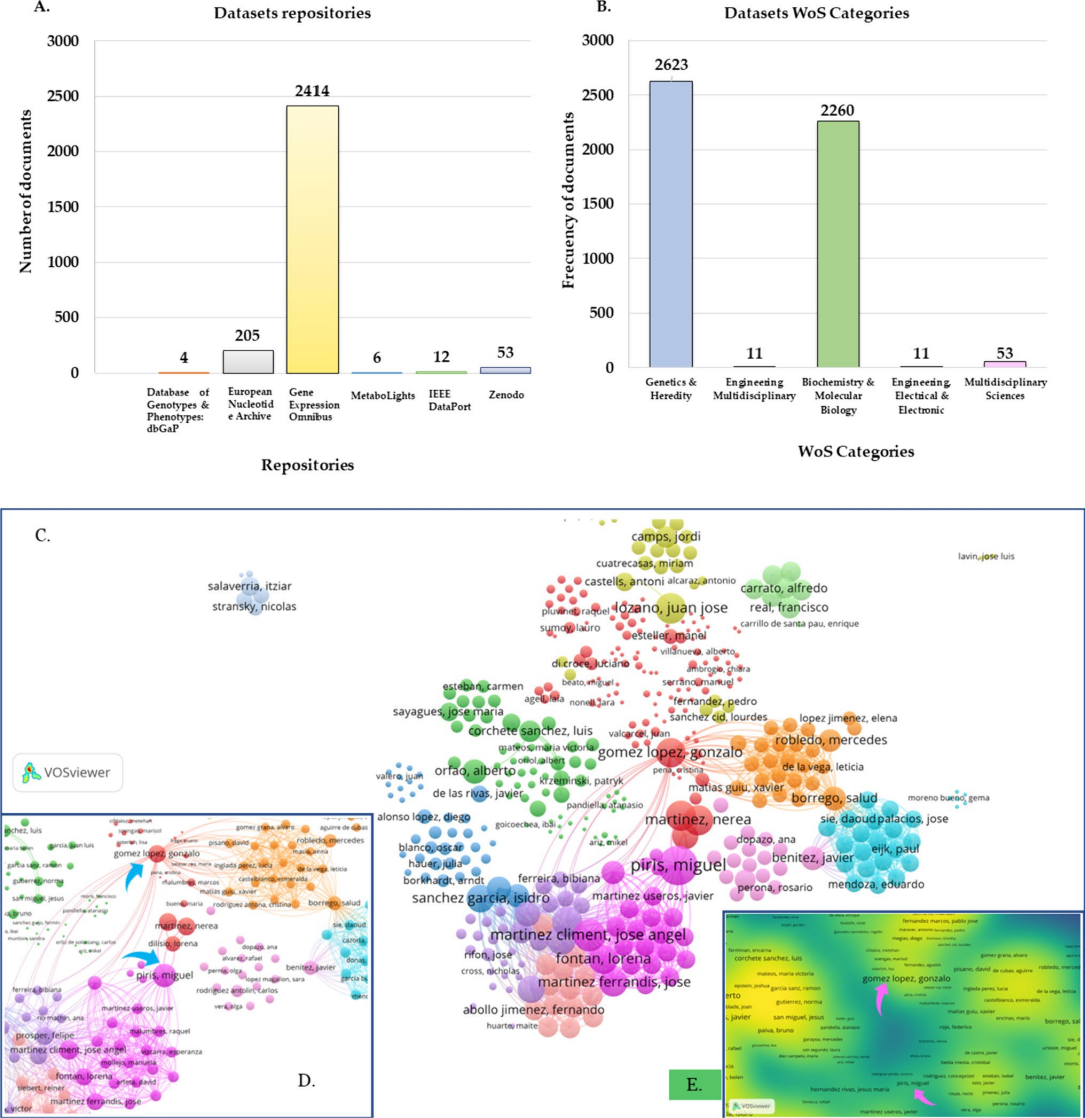
Analysis of the oncology datasets deposited by at least one Spanish author during 2011–2021, in **a** the different repositories; **b** distribution of repositories through the WoS categories; **c** authorship network related to data sharing practices in oncology research (2011–2021); **d** extension of Fig. 5c; **e** density visualization network of the number of links between authors

A total of 2693 records were retrieved from the DCI. An analysis of the repositories showed that the deposited data were mainly genetic in nature, with Gene Expression Omnibus standing out, followed by European Nucleotide Archive and Zenodo, with 2414, 205 and 53 datasets, respectively (Fig. 5a). These results are corroborated by the WoS subject categories of the repositories where the datasets were deposited: Genetics & Heredity (2623 datasets), Biochemistry & Molecular Biology (2260 datasets) and Multidisciplinary Sciences (53 datasets) (Fig. 5b).

The analysis of the citations received by the datasets of each repository placed in first position of the ranking Gene Expression Omnibus repository (309 received cites), followed by European Nucleotide Archive (70 received cites) and Database of Genotypes and Phenotypes: dbGaP (7 received cites), however, the datasets deposited in IEEE Dataport did not receive any citations. Regarding the relationships established between the most frequent authors in the practice of data deposition, Piris, Miguel stood out with 307 records and links to 33 authors. On the other hand, Gómez López, Gonzalo (183 records) had the highest number of links with other authors (*n* = 78) (Fig. 5c–e).

## Discussion

The aim of this study was to address the state of the art on cancer research with Spanish participation and its relationship with open science, both at the level of publications and research data dissemination. Numerous papers have been found in the literature based on the use of bibliometric techniques to investigate research on different types of cancer, however, this study relates for the first time the evaluation of oncological research production under the Open Science scenario, analysing its impact through received citations and international collaborations between researchers.

The 50,822 publications retrieved, and the 2693 datasets obtained showed, in addition to the increment of scientific production in cancer with Spanish participation, the relevance of detected open access articles and data sharing during 2011–2021.

It should be highlighted that the number of papers in 2021 has doubled compared to 2011, which could be related to the shocking data on the prevalence of cancer both in Spain and worldwide, and the effort being made by all institutions to promote basic and translational cancer research. Along with the significant rise in the number of publications, there has been an increase in the OA publications. This OA modality has steadily grown over the past decade and now comprises over half of the total publications in the most recent year studied. This means that cancer research with Spanish participation has not been alien to the commitment of the scientific community to Open Access, which crystallized two decades ago with the publication of the so-called three “B’s” (Budapest, Bethesda and Berlin Declaration), that have celebrated their 20th anniversary this year 2023 [14], and has been evidenced over time by the requirements of numerous institutions to Open Science [7, 15]. Observing the studied decade, it is in 2014 when the trend towards publishing in Open Access journals was produced, which is not surprising in the European context, since it was precisely that year when the H2020 projects (in force from 2014–2020) established the open publication as a precept of the works derived from funded projects, a trail followed by the Member States in their national policies.

The evolution observed in our results, is also consistent with the recent creation of the Coalition for Advancing Research Assessment (CoARA) in Europe, by which more than 400 institutions commit to supporting OS, including both data and publications and to recognize this commitment as a merit to the research staff [16].

Although it is true that the predominant access via is the Green route specially in the early years of the study (by which the author deposits the postprint of an article in a repository, once the embargo period has expired), a notable modification in the trend is detected. This change shows information that is key for understanding the relevance of OA in the current context in cancer research, which is represented by the large increase in publications in the so-called Golden way (public, immediate, permanent and free access to the final article for readers, usually after payment of APC—article processing charge—by the authors), and the decrease in the Bronze way (only access to reading the work, but without any type of open license). Due to its characteristics, the golden way is defended in the framework of open science, since it represents the essence of the OA philosophy in terms of the opportunity it offers for immediacy, access and reuse, elements that are even more important in an area such as cancer research [17]. Alongside its benefits, potential drawbacks or adverse consequences of the Gold model have also been recognized. In the studied period, there has been a tendency to disseminate cancer articles in journals of publishers that publish solely and exclusively in open access by paying APC. In particular, controversial issues relating to the APC model have been cited, as it has the potential to place authors at the forefront of journals’ commerce, resulting in a reduction in the standard of quality criteria to favour quantity and increase revenue [18].

However, this argument of declining quality is not supported by our study. In fact, our results have shown, both in terms of weighted citations received by the articles and JIF quartile distributions of the journals, that papers published through the OA modalities obtain better scores than those non-OA. This result agrees with other studies such as that of Gumpenberger et al. (2013) [19], that almost a decade ago, when there were still fewer OA journals, did already mention the positive relationship between Gold OA and positive impact, as well as the fact that open articles published were more cited comparing to other OA models. On this specific aspect, there are numerous papers that have investigated the positive impact in terms of citations received from OA [20]. It is interesting to highlight the study of Levin et al. (2023) [21] and AlRyalat et al. (2019) [22], since both examine the case of oncology research and OA publications and conclude that the number of citations received is higher when the publications are open, either in Gold OA journals or in Hybrid journals. Regarding collaborations, our study show that international collaborations are not only beneficial because they promote diversity of views among researchers and countries when it comes to research and therapeutic approaches [23]; but they also (or because of this) generate higher impact publications, information that is consistent with studies such as that of Kohus et al. (2022) [24]; moreover, within the publications with international collaboration, those that are in OA have an even greater presence in Q1 journals.

Regarding the practice of data sharing, it has historically followed a different trajectory than open publishing. While open publications belong to the very specific context of the OA movement of the late 1990s and early 2000s, the practice of sharing data among researchers is much older [25]. The way in which the practice of data sharing has been included in the orbit of open science has changed, making it more than just sharing limited to “face to face” researchers [26]. In the case of the research data in our study they are mostly genetics and molecular biology related data and were deposited in the Gene expression Omnibus repository, followed by far by the European Nucleotide Archive. The area of oncology has an important basic research component and, within this, genetics, which proved to be the type of research within the field of health sciences in which the greatest number of datasets are shared, and with a greater culture of data sharing [27, 28]. An example of the relevance is found in the study of Birney et al. (2017) [29], where they refer that, by 2030, 83 million genomes of rare diseases will be sequenced, as well as almost 250 million for cancer diagnosis. Another interesting work on the relationship between genetic data, data sharing practice and cancer research is that of Knoppers & Joly (2018) [30], that after reviewing two initiatives related to shared data in health, emphasizes that if there are no common policies and genomic data are not linked to daily clinical practice through coordinated and interoperable systems, it is difficult to improve clinical decisions.

## Limitations

The works retrieved through WoS SCIE and DCI represent the total of existing publications and datasets derived from journals or repositories, respectively, that have been indexed in these databases.

## Conclusions

Our study shows the growth in the number of cancer publications in the last decade, developed by at least one researcher from a Spanish institution, accompanied by an increase in the habit of sharing their studies, which marks a change in the attitude of cancer researchers towards a more open stance and closer to open science. In addition, the approach to more accessible OA models provides the opportunity to generate higher impact publications, just as it does when conducted between international research teams. Regarding research data, it has been observed that genetics area has the greatest sharing, which is a positive aspect that allows further progress in this line. However, oncology research is much more than genetics; there is a whole field of clinical and even social research that does not seem to be sharing research data at the same rate, therefore it would be very interesting to find out more about why this is happening. On the other hand, our study has allowed us to know what is shared in terms of repositories and the thematic categories where belongs, but it would be very interesting to delve more deeply into the content and structure of these datasets and their metadata in a more qualitative way, as well as to inquire about their quality in relation to compliance with the FAIR principles.

## Supplementary Information

Below is the link to the electronic supplementary material.

Supplementary file1 (DOCX 69 KB)

Supplementary file2 (DOCX 16 KB)

## Data Availability

The data generated and used in this study are openly available from the Zenodo.org public repository at https://zenodo.org/records/10101971.

## References

[1] World Cancer Day 2023: Close the care gap – PAHO/WHO | Pan American Health Organization [Internet]. [cited 2023 Nov 6]. Available from: https://www.paho.org/en/campaigns/world-cancer-day-2023-close-care-gap.

[2] GLOBOCAN 2020: New Global Cancer Data | UICC [Internet]. [cited 2023 Nov 8]. Available from: https://www.uicc.org/news/globocan-2020-new-global-cancer-data.

[3] Cancer cases and deaths on the rise in the EU [Internet]. 2023 [cited 2023 Nov 6]. Available from: https://joint-research-centre.ec.europa.eu/jrc-news-and-updates/cancer-cases-and-deaths-rise-eu-2023-10-02_en.

[4] El cáncer en cifras | SEOM: Sociedad Española de Oncología Médica [Internet]. [cited 2023 Nov 6]. Available from: https://seom.org/publicaciones/el-cancer-en-espanya.com.

[5] News | Redecan [Internet]. [cited 2023 Nov 6]. Available from: https://redecan.org/en/news/29/redecan-publishes-the-estimates-of-the-incidence-of-cancer-in-spain-2023.

[6] Berlin Declaration [Internet]. [cited 2023 Nov 8]. Available from: https://openaccess.mpg.de/Berlin-Declaration.

[7] Data sharing and management policy [Internet]. Cancer Res. UK. 2014 [cited 2023 Nov 6]. Available from: https://www.cancerresearchuk.org/funding-for-researchers/applying-for-funding/policies-that-affect-your-grant/data-sharing-and-management-policy.

[8] Ross JS, Ritchie JD, Finn E, Desai NR, Lehman RL, Krumholz HM, et al. Data sharing through an NIH central database repository: a cross-sectional survey of BioLINCC users. BMJ Open. 2016;6: e012769. 27670522 10.1136/bmjopen-2016-012769 PMC5051517

[9] Pasquetto IV, Randles BM, Borgman CL. On the reuse of scientific data. Data Sci J. 2017;16:8.

[10] Data Sharing and Public Access Policies | CBIIT [Internet]. [cited 2023 Nov 6]. Available from: https://datascience.cancer.gov/data-sharing/policies.

[11] Data Management & Sharing Policy Overview | Data Sharing [Internet]. [cited 2023 Nov 6]. Available from: https://sharing.nih.gov/data-management-and-sharing-policy/about-data-management-and-sharing-policies/data-management-and-sharing-policy-overview#before.

[12] Lawlor RT. The impact of GDPR on data sharing for European cancer research. Lancet Oncol. 2023;24:6–8. 36400103 10.1016/S1470-2045(22)00653-2

[13] Open Access WoS [Internet]. [cited 2023 Nov 6]. Available from: https://webofscience.help.clarivate.com/en-us/Content/open-access.html.

[14] Open Access [Internet]. [cited 2023 Nov 9]. Available from: https://www.mpifg.de/1006829/open-access.

[15] Anglada L, Abadal E. Open access: a journey from impossible to probable, but still uncertain. Prof Inf [Internet]. 2023 [cited 2023 Nov 9];32. Available from: https://revista.profesionaldelainformacion.com/index.php/EPI/article/view/87260.

[16] COARA 2022_07_19_rra_agreement_final.pdf [Internet]. [cited 2023 Nov 28]. Available from: https://coara.eu/app/uploads/2022/09/2022_07_19_rra_agreement_final.pdf.

[17] van der Heyden MAG, van Veen TAB. Gold open access: the best of both worlds. Neth Heart J. 2018;26:3–4. 29196877 10.1007/s12471-017-1064-2 PMC5758455

[18] Torres-Salinas D, Robinson-García N, Moed HF. Disentangling Gold Open Access. In: Glänzel W, Moed HF, Schmoch U, Thelwall M, editors. Springer handbook of science and technology indicators [Internet]. Cham: Springer International Publishing; 2019 [cited 2023 Nov 9]. p. 129–44. Available from: 10.1007/978-3-030-02511-3_5..

[19] Gumpenberger C, Ovalle-Perandones M-A, Gorraiz J. On the impact of Gold Open Access journals. Scientometrics. 2013;96:221–38.

[20] Richardson SL, Hedrick SG. Journal impact factors and the future of open access publishing. J Appl Clin Med Phys. 2023;24: e14083. 37357568 10.1002/acm2.14083 PMC10338788

[21] Levin G, Harrison R, Ledermann J, Meyer R, Coleman RL, Ramirez PT. Evaluating open access publication and research impact in gynecologic oncology. Int J Gynecol Cancer [Internet]. 2023 [cited 2023 Nov 9];33. Available from: https://ijgc.bmj.com/content/33/7/1112. 10.1136/ijgc-2023-004460 37220951

[22] AlRyalat SA, Nassar AA, Tamimi F, Al-Fraihat E, Assaf L, Ghareeb R, et al. The impact of the open-access status on journal indices: oncology journals. J Gastrointest Oncol [Internet]. 2019 [cited 2023 Nov 9];10. Available from: https://jgo.amegroups.org/article/view/27415. 10.21037/jgo.2019.02.13 PMC6657315 31392058

[23] Cree IA, Indave Ruiz BI, Zavadil J, McKay J, Olivier M, Kozlakidis Z, et al. The international collaboration for cancer classification and research. Int J Cancer. 2021;148:560–71. 32818326 10.1002/ijc.33260 PMC7756795

[24] Kohus Z, Demeter M, Szigeti GP, Kun L, Lukács E, Czakó K. The influence of international collaboration on the scientific impact in V4 countries. Publications. 2022;10:35.

[25] Sieber J. Data sharing in historical perspective. J Empir Res Hum Res Ethics. 2015;10:215–6. 26297743 10.1177/1556264615594607

[26] Popkin G. Data sharing and how it can benefit your scientific career. Nature. 2019;569:445–7. 31081499 10.1038/d41586-019-01506-x

[27] Mighton C, Smith AC, Mayers J, Tomaszewski R, Taylor S, Hume S, et al. Data sharing to improve concordance in variant interpretation across laboratories: results from the Canadian Open Genetics Repository. J Med Genet. 2022;59:571–8. 33875564 10.1136/jmedgenet-2021-107738 PMC8523590

[28] Vassilakopoulou P, Aanestad M. Communal data work: data sharing and re-use in clinical genetics. Health Informatics J. 2019;25:511–25. 30887878 10.1177/1460458219833117

[29] Birney E, Vamathevan J, Goodhand P. Genomics in healthcare: GA4GH looks to 2022 [Internet]. bioRxiv; 2017 [cited 2023 Nov 9]. p. 203554. Available from: https://www.biorxiv.org/content/10.1101/203554v1.

[30] Knoppers BM, Joly Y. Introduction: the why and whither of genomic data sharing. Hum Genet. 2018;137:569–74. 30097718 10.1007/s00439-018-1923-y

