# Supplementary figures and images for: State of open science in cancer research
Source: Clin Transl Oncol. 2024 Apr 18;26(10):2457–65. doi: 10.1007/s12094-024-03468-7 (PMC11410906; doi:10.1007/s12094-024-03468-7)

Supplementary Figure 1


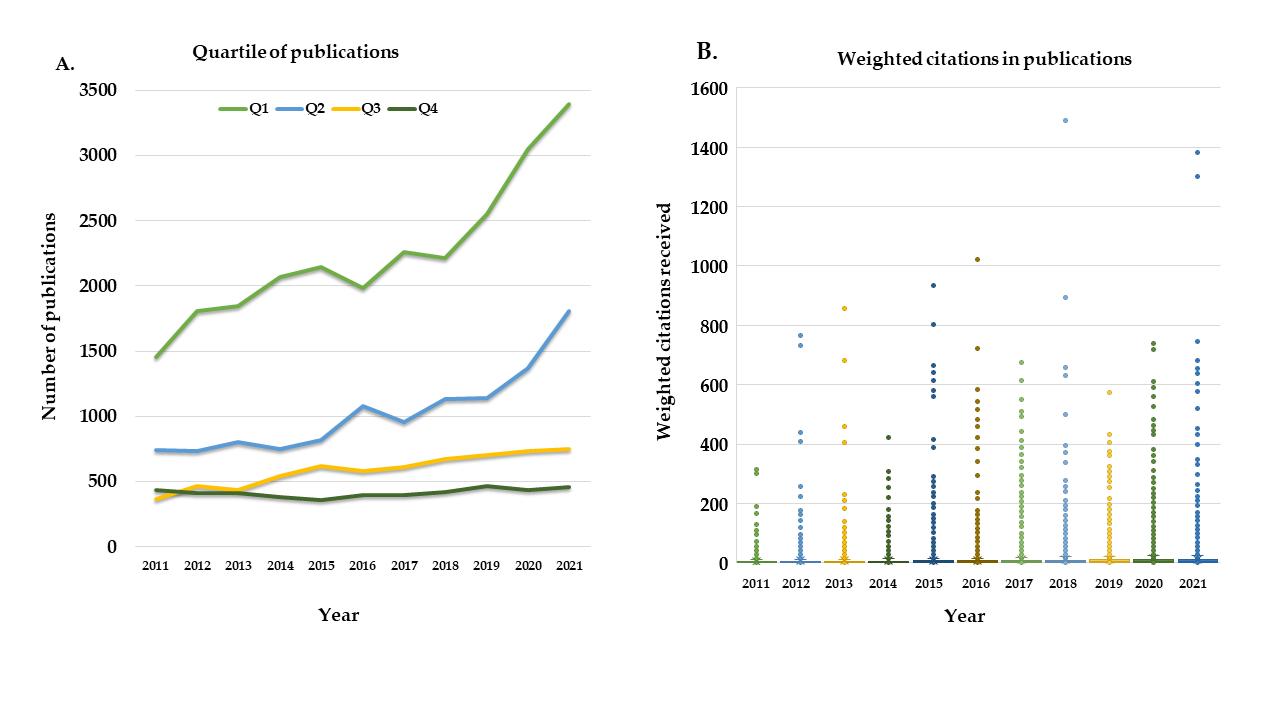

Supplement: Supplementary file 1 — Supplementary file1 (DOCX 69 KB) [file 12094_2024_3468_MOESM1_ESM.docx]
